# Identification of patients with nonischemic dilated cardiomyopathy at risk of malignant ventricular arrhythmias: insights from cardiac magnetic resonance feature tracking

**DOI:** 10.1186/s12872-023-03655-4

**Published:** 2024-01-03

**Authors:** Hai-Yan Ma, Guang-You Xie, Jian Tao, Zong-Zhuang Li, Pan Liu, Xing-Ju Zheng, Rong-Pin Wang

**Affiliations:** 1 Department of Radiology, Guizhou Provincial People’s Hospital, Guiyang, 550002 China; 2 Department of Cardiology, Guizhou Provincial People’s Hospital, Guiyang, 550002 China

**Keywords:** Cardiac magnetic resonance feature tracking, Dilated cardiomyopathy, Ventricular arrhythmias

## Abstract

**Background:**

Patients with nonischemic dilated cardiomyopathy (NIDCM) are prone to arrhythmias, and the cause of mortality in these patients is either end-organ dysfunction due to pump failure or malignant arrhythmia-related death. However, the identification of patients with NIDCM at risk of malignant ventricular arrhythmias (VAs) is challenging in clinical practice. The aim of this study was to evaluate whether cardiovascular magnetic resonance feature tracking (CMR-FT) could help in the identification of patients with NIDCM at risk of malignant VAs.

**Methods:**

A total of 263 NIDCM patients who underwent CMR, 24-hour Holter electrocardiography (ECG) and inpatient ECG were retrospectively evaluated. The patients with NIDCM were allocated to two subgroups: NIDCM with VAs and NIDCM without VAs. From CMR-FT, the global peak radial strain (GPRS), global longitudinal strain (GPLS), and global peak circumferential strain (GPCS) were calculated from the left ventricle (LV) model. We investigated the possible predictors of NIDCM combined with VAs by univariate and multivariate logistic regression analyses.

**Results:**

The percent LGE (15.51 ± 3.30 vs. 9.62 ± 2.18, P < 0.001) was higher in NIDCM patients with VAs than in NIDCM patients without VAs. Furthermore, the NIDCM patients complicated with VAs had significantly lower GPCS than the NIDCM patients without VAs (− 5.38 (− 7.50, − 4.22) vs.−9.22 (− 10.73, − 8.19), P < 0.01). Subgroup analysis based on LGE negativity showed that NIDCM patients complicated with VAs had significantly lower GPRS, GPCS, and GPLS than NIDCM patients without VAs (P < 0.05 for all). Multivariate analysis showed that both GPCS and %LGE were independent predictors of NIDCM combined with VAs.

**Conclusions:**

CMR global strain can be used to identify NIDCM patients complicated with VAs early, specifically when LGE is not present. GPCS < − 13.19% and %LGE > 10.37% are independent predictors of NIDCM combined with VAs.

## Introduction

Nonischaemic dilated cardiomyopathy (NIDCM) is characterized by systolic dysfunction and dilatation of the left ventricle (LV) in the absence of coronary artery disease or abnormal loading conditions [1]. In clinical practice, patients with NIDCM are prone to arrhythmias, and the cause of mortality in these patients is either end-organ dysfunction due to pump failure or malignant arrhythmia-related death [2]. Thus, an accurate clinical assessment of patients with NIDCM is crucial to identify those who are more likely to experience malignant ventricular arrhythmias (VAs). However, the identification of patients with NIDCM at risk of malignant VAs is challenging in clinical practice. From several studies analysing the substrate for Vas, scholars have reported that myocardial fibrosis plays an important role in the genesis of ventricular arrhythmias in patients with NIDCM [3–7]. Therefore, patients with NIDCM combined with malignant VAs should be continuously reassessed, particularly in the presence of abrupt worsening of left ventricle (LV) function or an increased VA burden.

At present, novel cardiovascular magnetic resonance (CMR) techniques are rapidly emerging as useful and unique tools for comprehensive cardiac evaluation of NIDCM, including chamber size quantification, evaluation of ventricular function and mass, myocardial wall thicknesses, segmental function, myocardial perfusion and fibrosis, myocardial oedema, and tissue characterization [8–10]. Compared with other indirect LV functional parameters, cardiovascular magnetic resonance feature tracking (CMR-FT) is a promising technique for the quantitative assessment of regional LV function and can be used for the early detection of subclinical myocardial abnormalities [11, 12]. Moreover, CMR-FT provides a broad field of view, superior image quality without limitations related to patient habitus or challenging acoustic windows, and a higher signal-to-noise ratio compared to speckle-tracking echocardiography. As such, CMR-FT could emerge as a compelling alternative to echocardiography for assessing the increasingly crucial parameters of myocardial deformation [13, 14].

Currently, only a limited number of studies have been designed to identify NIDCM patients at risk of VAs using CMR-FT [15]. However, due to variations in research objectives, methodologies, and sample sizes, the utility of magnetic resonance tissue tracking in assessing NIDCM patients with associated ventricular arrhythmias warrants further validation. Therefore, the purpose of the current study was to investigate the left ventricular myocardial strain and study potential predictors of NIDCM combined with VAs by using CMR-FT.

## Methods

### Patient population

**Fig. 1:**
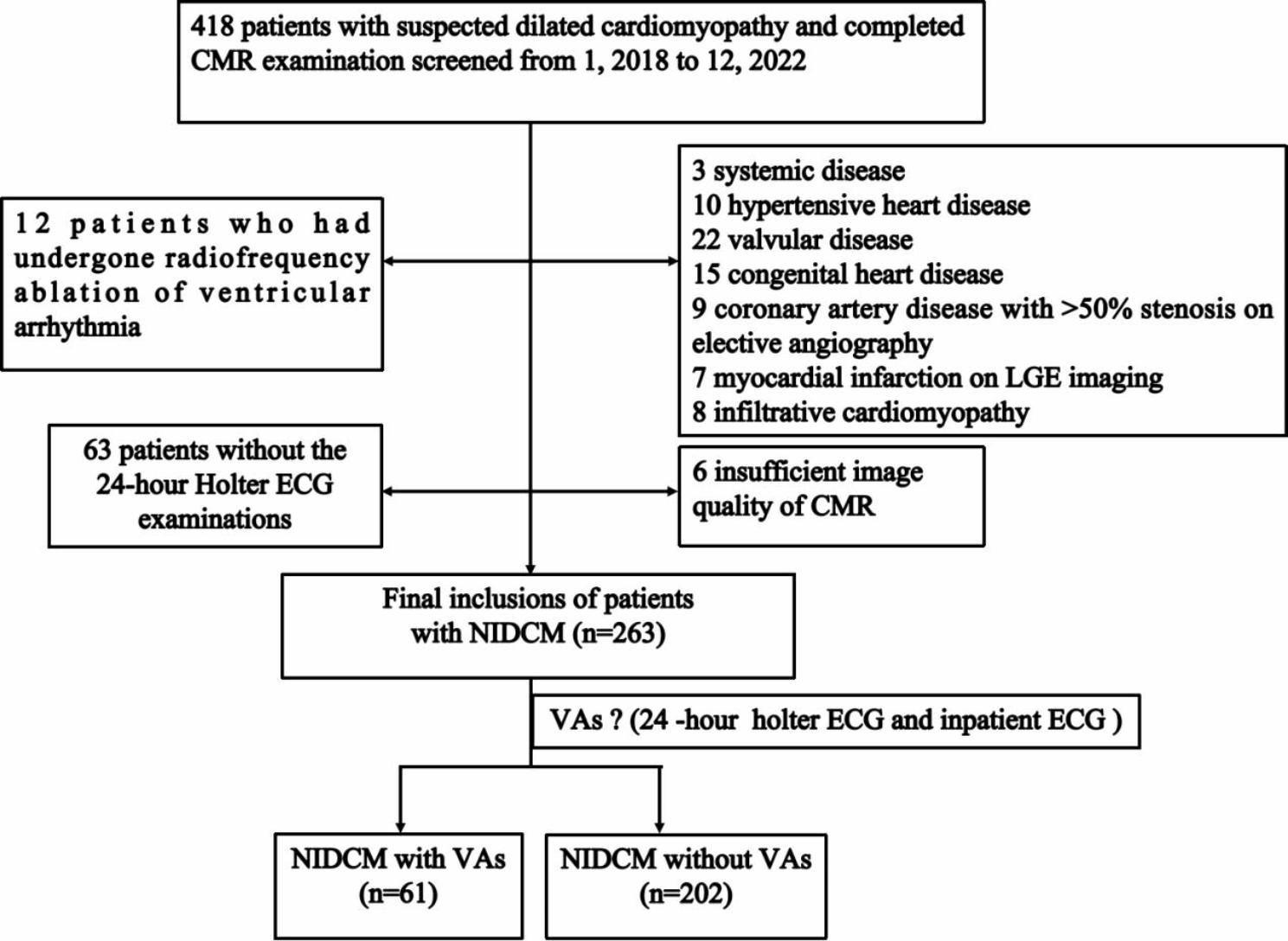
Flow chart shows selection of patients with NIDCM for the study. NIDCM, nonischemic dilated cardiomyopathy; CMR, cardiac magnetic resonance; VAs, ventricular arrhythmias; ECG, electrocardiography

We performed a retrospective study in a cohort of 418 consecutive patients with suspected NIDCM who had undergone CMR examination in our hospital between January 2018 and December 2022 (Fig. 1). **The inclusion criteria were as follows**: (i) the definition and diagnosis of patients with NIDCM was made according to the CMR current recommendations [16]; (ii) NIDCM patients underwent both CMR, 24-hour Holter electrocardiography (ECG) and inpatient ECG within a one-week period as well as other relevant assistant examinations (such as coronary angiography and echocardiography). **The exclusion criteria were as follows**: (i) coronary artery disease with > 50% stenosis on elective angiography; (ii) patients who had undergone radiofrequency ablation of ventricular arrhythmia; (iii) systemic disease; (iv) hypertensive heart disease; (v) valvular disease; (vi) congenital heart disease; (vii) previous myocardial infarction; (viii) infiltrative cardiomyopathy; and (viiii) inadequate images. In our study, patients included were not treated with ARNi and SGLT2I medications and were not included in multivariate regression analyses.

### Twenty-four-hour Holter ECG monitoring and image analysis

Malignant VAs defined as clinical presented as haemodynamic disorders and ECG showed malignant premature ventricular rhythm, paroxysmal ventricular tachycardia, persistent ventricular tachycardia, torsive ventricular tachycardia, ventricular flutter or ventricular fibrillation. Malignant ventricular premature beats refer to ventricular premature beats occurring more than 10,000 times within a 24-hour period. Even in the absence of obvious clinical discomfort, haemodynamic disturbances occur.

Three-channel 24-hour Holter ECG recordings were obtained for all of the patients in the absence of class I or class III antiarrhythmic drugs. The Holter recordings were analysed by a computer (DMS, USA) with manual overreading performed by two experienced cardiologists who were fully blinded to the clinical outcomes and CMR results. If there was a discrepancy between the two reviewers, a third senior reviewer was invited to make the final judgement.

### Cardiac magnetic resonance (CMR) imaging

CMR imaging was performed using a 1.5-Tesla (Aera, Siemens, Germany) and 1.5-Tesla (Verio, Siemens, Germany) following a standardized imaging protocol [17]. Balanced steady-state free precession (SSFP) sequences with breath-hold were performed to obtain cine CMR images, comprising a stack of contiguous parallel short-axis slices covering the entire LV from the base to the apex and three LV long-axis slice (2-, 3-, and 4-chamber views) images. SSFP sequence (repetition time/echo time/flip angle [TR/TE/FA] 2.4/1.2/50–75 degrees, matrix 192 × 256, resolution 1.9 × 1.9 × 8 mm^3^, field of view 289 mm×356 mm, temporal resolution ≤ 40 ms). Delayed enhancement images were acquired at end-diastole during breath holding using a segmented inversion-recovery gradient-echo turbo fast low-angle shot sequence obtained 8–10 min after the injection of 0.2 mmol/kg gadopentetate dimeglumine (Magnevist, Bayer) contrast medium. For the acquisition of all data, an 18-channel phased-array chest coil was used.

### CMR analysis

CMR feature-tracking (CMR-FT) analysis was performed using dedicated software (CVI42 v5.11, Circle Cardiovascular Imaging, Calgary, Canada). SAX and LAX cine images were uploaded into the software, which reconstructed a 3D model and derived peak radial, circumferential, and longitudinal strains. The LV strain was divided into a 17-segment model in accordance with the American Heart Association (AHA) [18]. The apical cap (segment 17) was not considered for analysis. In addition, the late gadolinium enhancement (LGE) mass and LV LGE percent were also quantitatively analysed using CVI42 software. The LGE was defined by using a signal intensity threshold level of 5 standard deviations (SDs) above a normal myocardial region that was used as a reference on the same section. LGE(+) means late gadolinium enhancement positive; LGE(-) means late gadolinium enhancement negative; %LGE means LGE(+) mass as the percentage of left ventricular myocardial mass. The same two cardiovascular radiologists who were blinded to the clinical histories independently analysed all CMR data.

### Statistical analysis

Statistical analysis was performed with commercially available statistical software (IBM SPSS Statistics 20.0, Chicago, Illinois). Qualitative or quantitative variables were expressed as the mean ± standard deviation (SD), median (interquartile range), and percentages as appropriate. Normal distribution was tested by the Kolmogorov–Smirnov test before analysing intergroup differences. For continuous variables, differences between two groups were obtained using unpaired Student’s *t* test or the Mann–Whitney *U* test. We investigated the association of different variables with NIDCM combined with VAs using univariate and multivariate logistic regression analyses. To address redundancy, which can lead to multicollinearity, we used statistical techniques such as the variance inflation factor (VIF) to assess the extent of multicollinearity among the variables. This might involve removing one of the highly correlated variables or employing other methods to mitigate multicollinearity while retaining the most important predictors in the model. Some baseline variables, such as the LGE percentage and GPCS strain, which were identified in univariate analysis as potential risk factors for NIDCM combined with VAs, were included in the multivariate logistic regression model. A logistic regression model was used to calculate the area under the receiver operating characteristic (ROC) curve for %LGE, GPCS strain, and %LGE combined with GPCS. The interobserver and intraobserver reproducibility for LV strain values were studied in a group of 26 randomly selected subjects by one observer, repeated twice, and by two observers who were unaware of each other’s measurements. The regression filling or multiple filling method was used for the missing data based on clinical and variable conditions. A two-tailed P < 0.05 value was considered statistically significant.

## Results

### Patient characteristics

Of the 418 consecutive patients initially enrolled in the study, 155 subjects were excluded based on the inclusion and exclusion criteria. Thus, 263 NIDCM patients (67% men; mean age: 49 years) were finally included. Furthermore, 263 patients with NIDCM were assigned to two subgroups based on 24-hour Holter examinations. There were 61 patients in the VA group and 202 patients in the non-VA group. There was no significant difference in patient characteristics (age, sex, body mass index) between the two groups (P > 0.05 for all), but the heart rate (beat/min) in the malignant VA group was significantly faster (109 ± 12 vs. 78 ± 13, P < 0.001) than that in the non-VA group.

In addition, the percent LGE (15.51 ± 3.30 vs. 9.62 ± 2.18, P < 0.001) was higher in NIDCM patients with VAs than in NIDCM patients without VAs. Furthermore, the NIDCM patients with VAs had significantly lower GPCS than the NIDCM patients without VAs (− 5.38(− 7.50, − 4.22) vs. −9.22 (− 10.73, − 8.19), P < 0.01). The results are summarized in Table 1.

**Table 1:** Baseline characteristics of the study population, analyzed separately according to the presence or absence of VAs

Variable	All Patients (n = 263)	NIDCM with VAs (n = 61)	NIDCM without VAs (n = 202)	t/χ^2^/Z	P value
Age (years)	49 ± 13	48 ± 14	49 ± 13	−0.49	0.62
Sex (male), n (%)	177 (67%)	38 (62%)	139 (69%)	0.01	0.93
BSA (m^2^)	1.76 (1.62, 1.88)	1.77 (1.47, 1.84)	1.75 (1.59, 1.82)	−0.11	0.61
Heart rate (beata/min)	94 ± 11	109 ± 12	78 ± 13	2.13	< 0.001
Family history, n (%)	51 (19%)	9 (15%)	42 (21%)	0.12	0.08
Diabetes, n (%)	31 (12%)	6 (10%)	25 (12%)	0.07	0.73
Hypertension, n (%)	68 (26%)	15 (25%)	53 (26%)	0.13	0.69
GFR (mL/min)	76 ± 7	74 ± 6	75 ± 8	−6.24	0.35
SCr (µmol/L)	90 ± 11	88 ± 6	89 ± 8	−5.17	0.42
**Medical treatment**					
β-blockers, n (%)	195 (74%)	48 (78%)	147 (73%)	2.76	0.04
Amiodarone, n (%)	6 (2%)	6 (10%)	—	—	—
ACE-inhibitors/ARBs, n (%)	203 (77%)	48 (78%)	155 (77%)	0.18	0.52
MRA, n (%)	111 (42%)	26 (43%)	85 (42%)	−3.74	0.32
Diuretic, n (%)	68 (26%)	16 (26%)	52 (26%)	0.36	0.78
Statin, n (%)	103 (39%)	24 (39%)	79 (39%)	0.18	0.69
**CMR parameters**					
LVEF (%)	41.61 ± 5.45	40.59 ± 7.83	41.75 ± 6.49	−4.18	0.07
LVEDVI (mL/m^2^)	189.55 (169.73, 212.34)	190.65 (164.05, 212.10)	188.40 (162.43, 210.81)	−0.76	0.09
LVESVI (mL/m^2^)	121.73 (104.56, 139.25)	125.15 (110.13, 133.35)	121.26 (101.67, 137.76)	−2.96	0.12
SVI (mL/m^2^)	40.33 ± 10.28	39.24 ± 9.66	40.19 ± 10.96	−0.29	0.76
CI (L/min/m^2^)	3.81 (2.71, 4.62)	3.70 (2.81, 4.53)	3.82 (2.63, 4.57)	−0.38	0.70
LGE (+), n (%)	132 (50%)	40 (66%)	92 (46%)	0.46	0.06
Percent LGE (%)	11.26 ± 2.35	15.51 ± 3.30	9.62 ± 2.18	10.39	< 0.001
GPRS (%)	11.46 (9.51, 15.44)	10.59 (8.44, 13.96)	11.66 (9.42, 15.21)	−2.44	0.09
GPCS (%)	−9.01 (− 11.05, − 8.23)	−5.38 (− 7.50, − 4.22)	−9.22 (− 10.73, − 8.19)	−5.56	< 0.01
GPLS (%)	−9.62 ± 2.32	−9.13 ± 2.25	−9.90 ± 2.07	3.64	0.05

Results are reported as mean ± SD, percentages, or median (interquartile range) as appropriate. NIDCM, nonischemic dilated cardiomyopathy; BSA, body surface area; GFR, glomerular filtration rate; SCr, serum creatinine; ARB, angiotensin receptor blocker; MRA, mineralocorticoid receptor antagonist; LVEF, left ventricular ejection; EDV, end-diastolic volume; ESV, end-systolic volume; SV, Stroke volume; CI, Cardiac index; LVEDVI, left ventricular end-diastolic volume index; LVESVI, left ventricular end-systolic volume index; SVI, indexed-stroke volume; LGE (+), late gadolinium enhancement positive; %LGE, LGE (+) mass as percentage of left ventricular myocardial mass; GPRS, global peak radial strain; GPCS, global peak circumferential strain; GPLS, global peak longitudinal strain

More importantly, based on the subgroup absence of LGE for NIDCM patients, the subgroup of NIDCM patients with VAs had no significant differences in LVEF, LVEDVI, LVESVI, SVI and CI compared with the subgroup of NIDCM patients without VAs (P > 0.05 for all; Table 2).

**Table 2:** Subgroup comparison CMR parameters of NIDCM patients combined with and without VAs based on the absence of LGE on CMR

Variable	NIDCM with VAs, LGE (-) (n = 21)	NIDCM without VAs LGE (-) (n = 110)	t/Z	P value
LVEF (%)	41.68 ± 7.25	42.89 ± 6.43	−5.42	0.07
LVEDVI (mL/m^2^)	191.65 (166.14, 213.23)	189.50 (167.75, 212.68)	−0.84	0.14
LVESVI (mL/m^2^)	120.76 (109.43, 140.65)	119.55 (101.67, 144.82)	−3.15	0.21
SVI (mL/m^2^)	40.24 ± 10.54	41.19 ± 11.75	−0.36	0.85
CI (L/min/m^2^)	3.70 (2.79, 4.63)	3.69 (2.33, 4.55)	−0.44	0.72
GPRS (%)	11.74 (9.23, 14.31)	14.66 (10.40, 17.45)	−4.31	0.02
GPCS (%)	−6.44 (− 8.26, − 5.15)	−10.33 (− 11.58, − 9.27)	−7.14	< 0.01
GPLS (%)	−9.75 ± 2.64	−11.85 ± 2.04	2.53	0.01

Results are reported as mean ± SD, percentages, or median (interquartile range) as appropriate. NIDCM, nonischemic dilated cardiomyopathy; LVEF, left ventricular ejection; CI, Cardiac index; LVEDVI, left ventricular end-diastolic volume index; LVESVI, left ventricular end-systolic volume index; SVI, indexed-stroke volume; LGE, late gadolinium enhancement; LGE (-), late gadolinium enhancement negative; GPRS, global peak radial strain; GPCS, global peak circumferential strain; GPLS, global peak longitudinal strain

### CMR global strain analysis

**Fig. 2:**
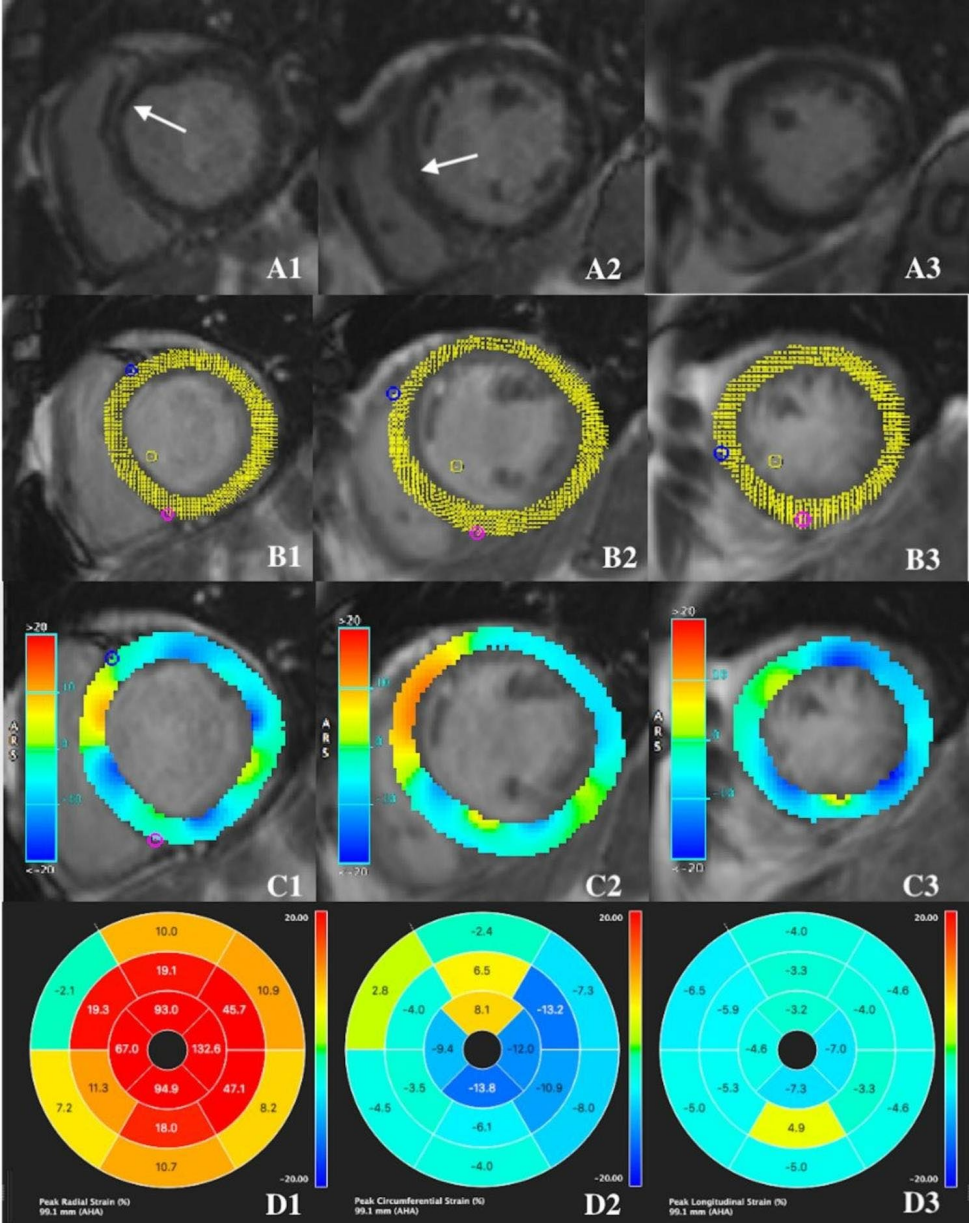
Representative example of the derivation of strain using cvi42 software in a 48-year-old male patient with NIDCM combined VAs. The result of LV feature-tracking of cine SSFP images in the short-axis view. Picture A1–3 The late gadolinium enhancement (LGE) on CMR shows the LGE positivity in the interventricular septal midwall of ventricular fibers in the basal, midventricular and apical short-axis view (white arrows). Picture B1-3: Myocardial strain feature-tracking are illustrated on basal, midventricular and apical short-axis view. Picture C1-3: Myocardial strain scale color map are illustrated on basal, midventricular and apical short-axis view. Picture D1-3: Strain of each segment on the bull’s eye diagram of AHA for the peak radial strain (%), peak circumferential strain (%), and peak longitudinal strain (%)

The NIDCM patients with VAs had significantly lower GPCS than those without VAs (− 5.38 (− 7.50, − 4.22) vs. −9.22 (− 10.73, − 8.19), P < 0.01). In contrast, compared with the NIDCM patients without VAs, the NIDCM patients with VAs exhibited no significant differences in GPRS (10.59 (8.44, 13.96) vs. 11.66 (9.42, 15.21), P > 0.05) and GPLS (− 9.13 ± 2.25 vs. −9.90 ± 2.07, P > 0.05; Table 1). A representative example of the derivation of strain in an NIDCM patient with VAs is shown in Fig. 2.

However, based on the subgroup absence of LGE for NIDCM patients, the subgroup of NIDCM patients with VAs had significantly lower in GPRS, GPCS, and GPLS compared with the subgroup of NIDCM patients without VAs (P < 0.05 for all; Table 2).

### Univariate and multivariate analyses of predictors of NIDCM combined with VAs

Multiple clinical and CMR parameters were included in the univariate analysis. Percent LGE and GPCS were all associated with NIDCM combined with VAs in univariate analysis (Table 3). After including two parameters in the multivariate analysis, percent LGE was shown to be the strongest predictor (odds ratio (95% CI) = 2.05 (1.50–2.81), P < 0.001) of NIDCM with VAs, followed by GPCS (odds ratio (95% CI) = 1.38 (1.14–1.67), P = 0.001; Table 3).

**Table 3:** Univariate and multivariate logistic regression analysis of predictors for NIDCM complicated with VAs

	Univariate logistic regression		Multivariate logistic regression	
	OR [95%CI]	p value	OR [95%CI]	p value
Age(years)	0.99 (0.96–1.02)	0.62	—	—
Sex	0.96 (0.40–2.30)	0.93	—	—
BSA (m^2^)	0.58 (0.07–4.83)	0.61	—	—
LVEF (%)	0.84 (0.78–0.91)	0.07	—	—
LVEDVI (mL/m^2^)	1.03 (1.01–1.04)	0.09	—	—
LVESVI (mL/m^2^)	0.94 (0.93–1.03)	0.12	—	—
SVI (mL/m^2^)	0.79 (0.68–1.02)	0.76	—	—
LGE (+)	0.91 (0.32–2.56)	0.06	—	—
LGE Percent (%)	1.92 (1.48–2.62)	< 0.001	2.05 (1.50–2.81)	< 0.001
GPRS (%)	0.64 (0.52–0.78)	0.09	—	—
GPCS (%)	1.43 (1.19–2.62)	< 0.01	1.38 (1.14–1.67)	0.001
GPLS (%)	0.97 (1.48–2.182)	0.05	—	—

BSA, body surface area; LVEF, left ventricular ejection; EDV, end-diastolic volume; ESV, end-systolic volume; LVEDVI, left ventricular end-diastolic volume index; LVESVI, left ventricular end systolic volume index; SVI, indexed-stroke volume; LGE (+), late gadolinium enhancement positive; %LGE, LGE (+) mass as percentage of left ventricular myocardial mass; GPRS, global peak radial strain; GPCS, global peak circumferential strain; GPLS, global peak longitudinal strain

### Diagnostic efficacy of percent LGE and GPCS strain for NIDCM combined with VAs

**Fig. 3:**
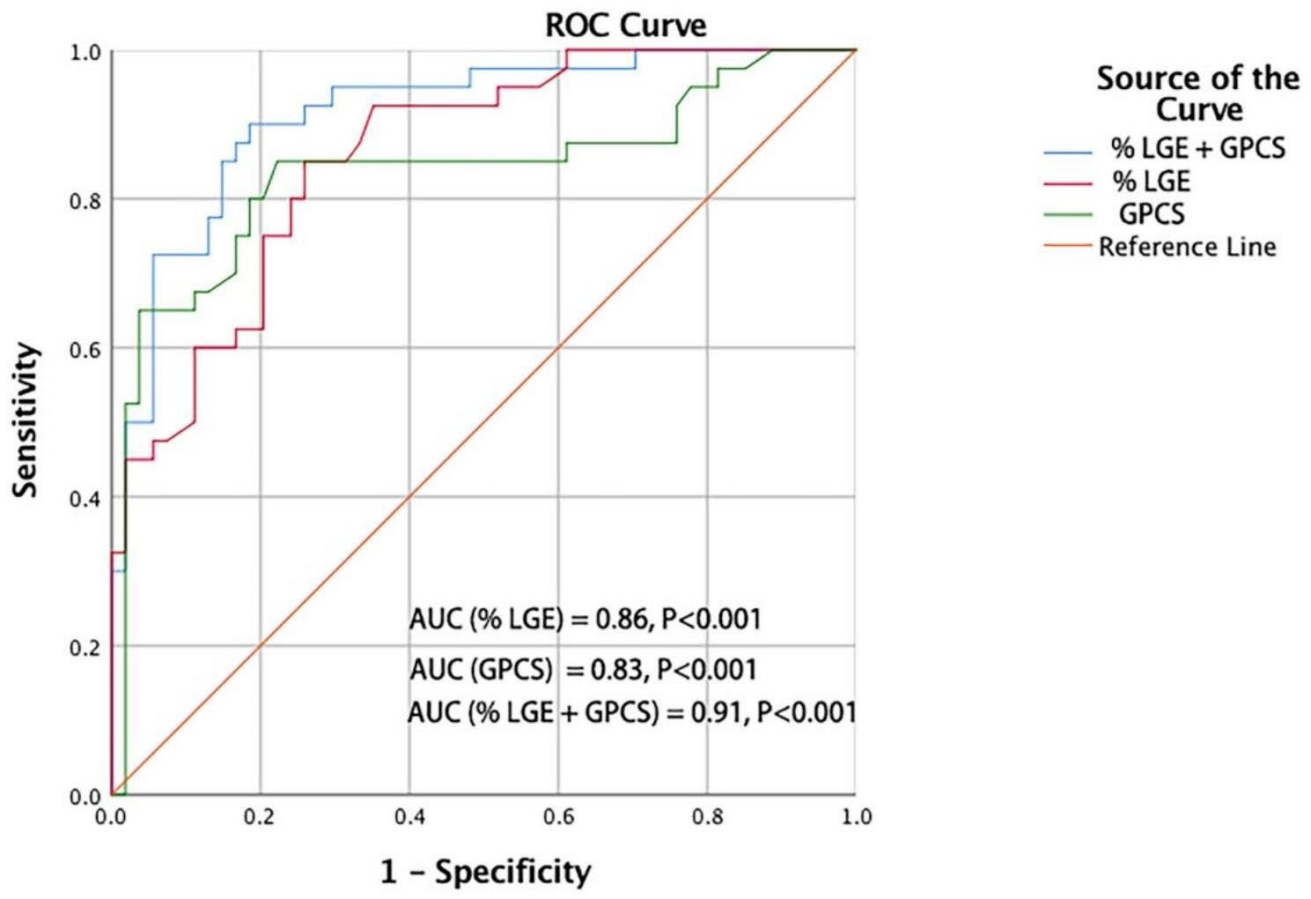
ROC curve analysis of %LGE values and GPCS for predicting on patients with NIDCM at risk of ventricular arrhythmias. %LGE, percent of LGE; GPCS, global peak circumferential strain; AUC, area under curve; ROC, receiver operator curve; NIDCM, nonischemic dilated cardiomyopathy

The ROC analysis of percent LGE and GPCS values showed a moderate discriminating capacity between NIDCM patients with and without VAs (AUC of LGE percent: 0.86, P < 0.001; AUC of GPCS: 0.83, P < 0.001) (Fig. 3). Furthermore, according to the ROC curve analysis, percent LGE combined with GPCS was the best parameter for identifying NIDCM combined with VAs (AUC of percent LGE + GPCS: 0.91, P < 0.001) (Fig. 3; Table 4).

**Table 4:** Diagnostic efficacy of LGE percentage and GPCS strain with respect to NIDCM combined with VAs

Variable	AUC	Optimal cut off	95%CI	Sensitivity (%)	Specificity (%)	PPV (%)	NPV (%)
LGE percent (%)	0.86	10.37	0.79–0.93	78.1%	83.0%	81.3%	80.5%
GPCS (%)	0.83	−13.19	0.75–0.92	76.4%	87.8%	84.2%	78.3%
LGE percent (%) + GPCS	0.91	—	0.86–0.97	81.0%	92.5%	90.4%	83.5%

LGE, late gadolinium enhancement; %LGE, LGE (+) mass as percentage of left ventricular myocardial mass; GPCS, global peak circumferential strain; PPV, positive predictive value; NPV, negative predictive value

Among these parameters, the specificity values of GPCS and percent LGE in distinguishing between NIDCM patients with and without VAs were 83.0% and 87.8%, respectively. The sensitivity values of GPCS and LGE in distinguishing between NIDCM patients with and without VAs were 78.1% and 76.4%, respectively. However, percent LGE combined with the GPCS value showed the highest sensitivity and specificity, with values of 81.0% and 92.5%, respectively, in distinguishing NIDCM patients with and without VAs. The optimal cutoff values of GPCS and %LGE for distinguishing patients with NIDCM combined with VAs from those without VAs were − 13.19% and 10.37%, respectively (Table 4).

### Reproducibility

Reproducibility of CMR-FT Parameters. Interobserver and intraobserver intraclass correlation coefficient (ICC) values in NIDCM patients for GPCS measurements were 0.934 and 0.937, respectively; for GPRS measurements, 0.841 and 0.913, respectively; and for GPLS measurements, 0.852 and 0.894, respectively (Table 5).

**Table 5:** Reproducibility of CMR-FT parameters

		Mean Difference		ICC (95% CI)		CoV (%)
Intraobserver						
GPLS		0.406 ± 1.092		0.894 (0.852–0.961)		5.84
GPCS		0.376 ± 0.785		0.937 (0.905–0.997)		4.15
GPRS		1.013 ± 1.526		0.913 (0.889–0.983)		7.71
Interobserver						
GPLS		1.328 ± 2.015		0.852 (0.693–0.907)		9.59
GPCS		0.270 ± 1.564		0.934 (0.903–0.995)		5.82
GPRS		0.516 ± 1.573		0.841 (0.809–0.941)		6.84

Values are mean ± SD; CI, confidence interval; CMR-FT, cardiac magnetic resonance myocardial feature tracking; CoV, coefficient of variation; GPLS, global peak longitudinal strain; GPCS, global peak circumferential strain; GPRS, global peak radial strain; ICC, intraclass correlation coefficient

## Discussion

Based on our findings, first, LV deformation analysis can differentiate NIDCM patients complicated with VAs early, specifically when LGE is not present or when VAs have been seen without a high %LGE. Second, LV GPCS and %LGE were independent predictors of NIDCM with VAs.

### Ventricular arrhythmias (VAs) and myocardial fibrosis

Patients with NIDCM are prone to arrhythmias. It is believed that the arrhythmogenic substrate results from myocardial fibrosis, leading to an “irritable focus” that is easily triggered [19]. In addition, a meta-analysis performed by Di Marco et al. [20] assessed the relationship between LGE and VAs in patients with NIDCM. Di Marco confirmed that the presence of LGE is strongly and independently associated with ventricular arrhythmias or sudden cardiac death. Furthermore, other studies have also shown that LGE is a better predictor of outcomes than age, LV volume, or LVEF, and it is an independent predictor of outcomes, even in less severely ill nonischemic DCM patients [21, 22]. Wu et al. [23] and Perazzolo Marra et al. [24] reported that the presence of LGE was associated with adverse cardiac events, regardless of the extent or pattern of LGE distribution. However, more recent work [25] proved that the predictive value of fibrosis was not specifically determined by its presence but by its extent and distribution.

Similar to the study by Halliday et al. [25], our research showed that the identification value of fibrosis was not specifically determined by its presence but by its extent (%LGE). In this study, we observed a significantly higher extent of %LGE in patients with NIDCM with VAs than in those without VAs. In contrast, there was no statistically significant difference in the presence of LGE between patients with NIDCM with VAs and without VAs. Therefore, a high %LGE (i.e., more extensive LGE distribution) detects the areas of myocardial fibrosis from which abnormal depolarization, unexcitable obstacles for wave propagation causing unidirectional blocks, and slow conduction favouring the development of reentry as a source of ventricular arrhythmias can originate [3, 26, 27].

### Ventricular arrhythmias (VAs) and left ventricular myocardial deformation

Previous studies have shown that LGE is the substrate for the occurrence of VAs, and VAs mainly occur in the areas of myocardial fibrosis of the dilated myocardium of NIDCM patients. The surrounding zone of the area of myocardial fibrosis is a heterogeneous medium where tissue with different levels of fibrosis coexists, resulting in both viable and nonviable myocardia. Myocardial fibrosis may constitute a substrate for VAs, where slow and heterogeneous conduction may favour the genesis of the reentry mechanism, thereby increasing the chance of developing malignant VAs [28–30].

Several other studies proved that classical NIDCM showed LGE positivity in nonischemic patterns with involvement of areas subjected to increased tension, such as the interventricular septal mid-wall at the site of insertion of ventricular fibres [31, 32]. Furthermore, several studies on LGE-CMR in patients with NIDCM have also shown typical mid-wall fibrosis [31], which is reparative microscopic scarring following myocyte death [33, 34]. Similarly, our study showed that patients with NIDCM with a higher %LGE in the myocardial mid-wall at the site of myocardial fibrosis were more prone to a decrease in GPCS.

However, only a small number of studies have used CMR-FT for the differential diagnosis of NIDCM patients with VAs [15]. In contrast to the results of the Linsheng Song [15] study, here we showed that GPCS was significantly lower in NIDCM patients with VAs than in NIDCM patients without VAs (− 5.38 (− 7.50, − 4.22) vs. −9.22 (− 10.73, − 8.19), P < 0.01). Of importance, our study also proved that specifically in subgroups of LGE-negative NIDCM patients, NIDCM patients with VAs had significantly lower GPRS, GPCS, and GPLS than NIDCM patients without VAs. Therefore, our study value is that CMR global strain can be used to identify NIDCM patients complicated with VAs early when LGE is not present.

Multivariate logistic analysis identified GPCS as an independent predictor of NIDCM combined with VAs (OR = 1.38, P = 0.001). Since NIDCM shows typical mid-wall fibrosis on CMR, it mainly affects the myocardial CS, leading to a decrease in GPCS in patients with NIDCM combined with VAs. Furthermore, another factor could also explain why GPCS alone was independently associated with patients with NIDCM at risk of VAs in our study. GPCS was found to be the feature tracking parameter with the highest reproducibility because it was not affected by poor tracking of the subannular region, unlike GPLS. For this reason, it was considered the most robust parameter in CMR-FT studies of myocardial strain [12, 35]. Finally, CMR-FT was different from strain echocardiography and strain-encoded MR on algorithms, and only numerical phantoms could give an absolute answer when evaluating different algorithms [36]. Therefore, patients with NIDCM with a higher %LGE in the myocardial mid-wall at the site of myocardial fibrosis and a decrease in GPCS are more prone to ventricular arrhythmias.

### Limitations

First, this was a single-centre observational study with a number of patients with NIDCM. Records were retrospectively collected, and patients were diagnosed as having NIDCM combined with ventricular arrhythmias. Second, we did not compare CMR-FT to other tracking modalities, such as speckle-tracking echocardiography, because previous studies compared CMR-FT with speckle-tracking echocardiography and proved the higher accuracy of CMR-FT [13, 14]. Third, the aim of this study was to evaluate whether CMR-FT could help in the identification of patients with NIDCM at risk of VAs, and we performed differential diagnosis for a subgroup of NIDCM; however, we did not conduct long-term clinical follow-up studies in NIDCM patients because it was not within the scope of our study. Fourth, we intended to exclude patients using β-blockers during Holter ECG recordings; however, due to the retrospective nature of the study, we cannot ensure complete exclusion. We anticipate that future prospective studies will provide a more robust assessment of β-blocker usage when analyzing the risk of ventricular arrhythmias in patients with NIDCM. Fifth, although the GPCS values are reproducible, many different factors may influence the quantification of LV strain, including image acquisition, algorithms, two different CMR platforms, and even different software programs [37], which is why the reported cutoff points are not applicable to other tools.

## Conclusions

LV global strains assessed by CMR-FT imaging were able to detect NIDCM combined with malignant VAs, specifically when LGE was not present. Global peak circumferential strain < − 13.19% and %LGE > 10.37% were independent predictors of NIDCM combined with VAs. The combination of GPCS and %LGE can be used to identify NIDCM patients at risk of VAs with high sensitivity and specificity.

## Data Availability

The data that support the findings of this study are available from the corresponding author upon reasonable request. All authors had complete access to the study data supporting this publication.

## Abbreviations

CMR: Cardiovascular magnetic resonance
CMR-FT: Cardiovascular magnetic resonance feature tracking
NIDCM: Nonischemic dilated cardiomyopathy
VAs: Ventricular arrhythmias
GPCS: Global peak circumferential strain
GPRS: Global peak radial strain
GPLS: Global peak longitudinal strain
LGE: Late gadolinium enhancement
LV: Left ventricle
LVEF: Left ventricular ejection fraction
LVEDVI: Left ventricular end-diastolic volume index
LVESVI: Left ventricular end-systolic volume index
ECG: Electrocardiography
